# Oral health literacy and oral health outcomes in an adult population in Brazil

**DOI:** 10.1186/s12889-017-4443-0

**Published:** 2017-07-26

**Authors:** Marília Jesus Batista, Herenia Procopio Lawrence, Maria da Luz Rosário de Sousa

**Affiliations:** 1 Community Health, Jundiai Medical School, Francisco Telles, 250, P.O. Box1295, Jundiaí, SP 13202-550 Brazil; 2 Community Health Graduate Program, Piracicaba Dental School, University of Campinas, Avenida Limeira 901, P.O. Box 52, Piracicaba, SP 13414-018 Brazil; 3 Dental Public Health Discipline, Faculty of Dentistry, University of Toronto, Canada, 124 Edward Street, Toronto, ON M5G 1G6 Canada; 4 Department of Community Dentistry, Piracicaba Dental School, University of Campinas, Brazil, Avenida Limeira 901, P.O. Box 52, Piracicaba, SP 13414-018 Brazil

**Keywords:** Adults, Oral health, Health literacy, Quality of life, Epidemiology, Logistic and multinomial regression analysis

## Abstract

**Background:**

To investigate the association between critical and communicative oral health literacy (OHL) and oral health outcomes (status, oral health-related quality of life and practices) in adults.

**Methods:**

This cross-sectional study examined a household probability sample of 248 adults, representing 149,635 residents (20–64 years old) in Piracicaba-SP, Brazil. Clinical oral health and socioeconomic and demographic data, as well as data on oral health-related quality of life (OHIP-14) and health practices were collected. The oral examinations were carried out in the participants’ homes, using the World Health Organization criteria for oral diseases. The critical and communicative OHL instrument was the primary independent variable, and it was measured using five Likert items that were dichotomized as ‘high’ (‘agree’ and ‘strongly agree’ responses for the 5 items) and ‘low’ OHL. Binary and multinomial logistic regressions were performed on each outcome (oral health status and practices), controlling for age, sex and socioeconomic status (SES).

**Results:**

Approximately 71.5% presented low OHL. When adjusted for age and sex (first model) low OHL was associated with untreated caries (Odds Ratio = 1.92, 95% Confidence Interval = 1.07–3.45), tooth brushing <3 times a day (OR = 2.00, 1.11–3.62) and irregular tooth flossing (OR = 2.17, 1.24–3.80). After SES inclusion in the first model, significant associations were found for low OHL when the outcomes were: presence of biofilm (OR = 1.83, 1.08–3.33), dental care for emergency only (OR = 2.24, 1.24–4.04) and prevalence of oral health impact on quality of life (OR = 2.06, 1.15–3.69).

**Conclusion:**

Adjusting for age, sex and SES, OHL is related to a risk factor (biofilm) and a consequence of poor oral health (emergency dental visits) and can interfere with the impact of oral diseases on quality of life. As low OHL can be modified, the results support oral health promotion strategies directed at improving critical and communicative oral health literacy in adult populations.

## Background

It is recognised that socioeconomic conditions are determinants of oral health, and in turn, oral health is an integral part of general health and quality of life [1]. Socioeconomic determinants and lifestyle choices, such as unhealthy eating, inadequate personal hygiene and lack of oral hygiene practices, inadequate sanitation, and insufficient exposure to fluorides, as well as tobacco and alcohol dependence, are health risk factors. The social and economic circumstances of individuals influence their behavioural choices, and consequently affect their health outcomes [2]. This fact makes it difficult for health professionals to control complex risk factors of health. However, to act on the causes of diseases it is fundamental to focus on health promotion with appropriate strategies [3]. Health literacy is a health promotion strategy that is one of the five key tracks identified at the 7th Global Conference on Health Promotion of the World Health Organization (WHO) [1].

Health literacy (HL) relates to the capacity of individuals to acquire, understand and take action on health information and make appropriate health decisions, with the final objective being the maintenance of their health or the management of a disease [4], which means autonomy in their health choices. The WHO defines health literacy as the ability to engage with health information and services in a meaningful way [5]. Thus, the concept of health literacy is wider than simply being able to read pamphlets and look for health services; it involves a set of skills needed to improve one’s capacity to use health information effectively, which means that health literacy is critical to empowerment [4]. Moreover, the concept of health literacy involves the ability to act politically to influence decision-making that will exert control over the social and economic determinants of health, which is a common omission among illiterate people [6].

Health literacy has been recognized as an important concept in counselling patients and in disease management [7]. Studies have shown that worse oral health outcomes have been associated with low health literacy and also lower use of health services [8]. A lower level of health literacy was found among adults who were more likely to miss their dental appointments [9]. For Horowitz and Kleinman [10], oral health literacy is paramount because low health literacy contributes to the spread of diseases, which results in increased costs to the population in general. Those with low oral health literacy are usually at the highest risk for oral diseases and the problems related to those diseases. Individuals with low health literacy include the poor, those with low levels of education, minorities and the elderly [10].

Conceptually, there are three types of literacy: 1) Functional literacy, which considers the reading and writing skills of a patient, such as understanding a prescription or dosage of a drug, having control over information on health risks and using health services; 2) Communicative/interactive literacy that evaluates the most advanced cognitive skills along with social skills, and addresses the ability to extract information from different media and apply new information to personal situations, thus promoting change in specific circumstances; and, 3) Critical literacy, which is the ability to critically analyse information and use that information to exercise greater control over life events and situations [4].

Ishkawa et al. [7] have created a brief instrument that assesses communicative and critical literacies using five questions in order to develop health promotion strategies for the population studied. The availability of information and the ability to acquire information from various sources of communication were assessed, as well as the ability to judge whether the information received is correct and if it can be applied to the health situation. For our study, we used the instrument of Ishkawa et al. [7] to assess the communicative and critical literacy of adults in Piracicaba city in the State of São Paulo, Brazil and their association with oral health outcomes, including oral health status measures, oral health-related quality of life (OHRQoL) and oral care practices. While we cannot modify the socioeconomic conditions that affect the health and oral health of individuals, oral health literacy can be modified and improved so as to serve as a strategy in the prevention and control of oral disease. The study hypotheses were that low oral health literacy is associated with worse oral health outcomes and greater impact on OHRQoL.

## Methods

### Study design and location

This was a cross-sectional survey carried out in the city of Piracicaba, State of São Paulo, Brazil, through household probability sampling. The Piracicaba population in 2010 comprised 368,836 residents and the adult population (20–64 years old) in the urban area included 170,611 residents at the time [11].

### Sample selection

This study is nested in a research project evaluating the oral health status of a cohort of 20 to 64 year-old adults who were residents of Piracicaba [12]. The sample size calculation was determined in order for the sample to be representative of the adult population of this municipality. The past caries experience was used as the calculation base, as measured by the DMFT (Decayed, Missed and Filled Teeth) index in adults [13], and adjusted for the population size of Piracicaba. An adjustment parameter was used for participants aged 20 to 44 years and another for those aged 45 to 64 years. We adopted the confidence interval of 95%, accuracy of 10% and design effect of 1.5. We added 30% to this total to compensate for possible losses and refusals, resulting in an estimated total sample of 240 adults.

The sample selection was conducted using probabilistic techniques, in two stages. At the first stage, the census tract served as the unit of selection, and 30 out of 456 census tracts (plus 2 in the case of a substitution) were selected based on the number of inhabitants in each unit. The second stage consisted in selecting the houses from each of the 30 census tracts, and we added 30% of the sample size to the randomly selected houses to compensate for the effects of non-response, thus resulting in a total of 342 houses. Based on the population size of each census tract, we randomly selected 11 houses per census tract, and then 1 adult per house was randomly selected. The inclusion criteria were: living in Piracicaba, being within the stipulated age range (20–64-years), having cognition to answer the questionnaire and agreeing to participate in the research. In order to avoid non-responses, the examiner returned up to three times to the house in case of absence of the adult volunteer randomly selected.

### Data collection

An examiner (one dentist) and a community health agent collected all the data between June and September 2011. Oral health examinations and questionnaires were carried out in the participants’ homes, which were located by means of a Piracicaba map and the census tract of the selected houses.

The calibration process included theoretical and practical discussions, and clinical oral examinations of volunteers. The process lasted eight hours until the dental examiner and the reference examiner reached at least 90% of concordance for caries and periodontal status [14]. An intra-observer agreement range from 96.5 to 100% was observed for caries and periodontal disease, and kappa was 0.89–1.00 and within the standards of excellent reliability [15].

Caries experience [Decayed, Missed, Filled Teeth index (DMFT)], periodontal disease [Community Periodontal Index (CPI)] and presence of biofilm [16] were the conditions assessed following the WHO criteria [14]. Demographic and socioeconomic data, as well as data on oral health literacy (OHL) [7], use of dental services, oral health practices and oral health-related quality of life (OHRQoL) [17] were obtained through a questionnaire. The instrument used to assess OHRQoL was the OHIP-14 [17], which had been validated in Brazil by Oliveira and Nadanovsky [18]. An interview was conducted by the examiner after the oral examinations had been completed using the structured questionnaire that contained 86 questions in total, part of which were from the 2010 SB Brazil [19] while the other items were tested via a pilot study.

### Data analysis

Data were analysed using the software Statistical Package for the Social Sciences (SPSS), version 19.0. Descriptive weighted analyses were performed so that we could obtain the population-adjusted frequency, mean, median and standard deviation (SD) of the variables which were the conditions examined.

The OHL was the main independent or explanatory variable, and it was measured using five Likert-scale items according to Ishikawa [7]. The five items were translated from English to Portuguese and adapted for the oral health context (Table 2). Questions 1 to 3 are related to communicative OHL, and 4 and 5 relate to critical OHL. The Likert-type response options were dichotomized for the analyses, meaning a score of ‘0’ was given to those who answered ‘agree’ or ‘strongly agree’, and ‘1’ to those who answered ‘do not agree or disagree’, ‘disagree’ or ‘strongly disagree.’ The responses for the five questions were then added together resulting in an overall OHL score, which could range from 0 to 5. It was considered ‘high’ OHL when all questions were answered with ‘agree’ or ‘strongly agree’, and ‘low’ OHL for those with at least one question ‘do not agree or disagree’, ‘disagree’ or ‘strongly disagree.’

Age was dichotomized into: 20 to 44 years and 45 to 64 years. Social Economic Status (SES) was measured according to a social classification created by Graciano et al. [20], which considers family income, number of residents per house, home ownership, adult occupation and adult education as factors that influence the inclusion in a given social class classification. A score is attributed to each criterion and the total score is calculated by summing all the scores, which are then classified into six social classes. For this study, the six classes of the variable SES were further re-grouped into three categories due to the skewness of the distribution: lower, lower-middle, and middle class or higher.

The oral health outcomes included:

- *Decayed teeth* or untreated caries as measured in the clinical exam using the DMFT Index (the D component of the index). It was considered ‘yes’ (or present) if the individual had at least one decayed teeth or ‘no’ (absence of decayed teeth);
- *Filled teeth* or the F component of the DMFT Index was considered ‘yes’ if the individual had at least one filled tooth or ‘no’ (absence of filled teeth);
- *Missing teeth* were operationalized using a Tooth Loss Classification (no missing teeth, missing up to 12 teeth including posterior teeth, missing up to 12 including anterior teeth, and missing 13 teeth or more) developed by Batista et al. [12];
- *Periodontal disease* was measured using the CPI, being considered ‘yes’ for those with at least one sextant presenting clinical attachment loss (CAL) more than 4 mm (codes 3 and 4 of the index);
- *Bleeding gums* were also measured using the CPI, being considered ‘yes’ for at least one sextant presenting bleeding, that is, the code 1 from the index;
- *Presence of dental plaque* (biofilm) according with Ainamo and Bay [16], considering ‘yes’ for at least one tooth surface with visible dental biofilm.
  Participants’ oral health practices, as measured during the interviews, were:
- *Tooth brushing*, dichotomized at twice a day or less, and 3 times or more per day;
- *Tooth flossing* was measured if the individual uses tooth floss every day, which was considered regularly, or some days per week, or per month, which were considered irregularly;
- *Frequency of use of dental services,* categorized as ‘regularly or every year’ or ‘sporadically,’ if services are used for emergency only;
- *Dental service evaluation* was considered for the last dental service used, being ‘not good’ (when the individual answered ‘bad,’ ‘regular’ and ‘very bad’) or ‘good’ (for ‘good’ and ‘very good’);
- *Reason for the use of dental services* was investigated if individuals had used dental services in the last visit due to pain or other urgent needs versus routine care, i.e., for a check-up;
- *Impact on OHRQoL* was assessed using the OHIP-14 ‘prevalence,’ which measures the proportion of individuals whose response was ‘fairly often/very often’ in at least one of the 14 scale items [12].

Other variables included: the service used in the last dental visit which was categorized as public, private, or health insurance; the time of the last appointment which was stratified into less than one year, between one and two years or three years or more, and; avoidance of dental treatment in the last year for any reason (yes or no).

In order to support our analyses, Macek’s conceptual model of health literacy and knowledge was adopted and further adapted to the oral health context. This model shows the importance of having these skills in making appropriate decisions regarding health (including oral health) that will impact on health outcomes [21].

Binary logistic regressions were performed when the dependent variables were dichotomous, and multinomial logistic regression was used for tooth loss and reason for the use of dental services, which are variables with more than two categories. The base model for each outcome was controlled for age and sex (male or female). In the second step, we included oral health literacy (model 1 in Tables 3 and 4) and, lastly, we included socioeconomic status (SES) in the final model (model 2 in Tables 3 and 4).

## Results

The respondents were 248 adults, representing a population residing in Piracicaba, Brazil, estimated at 149,325 adults aged 20 to 64 years. Most of those examined were females (72.2%, *n* = 179) and 55.6% (*n* = 138) were 20–44 years old. Regarding socioeconomic status, 15.3% (*n* = 38) belonged to the lower social class.

Low OHL presented greater prevalence among those who were classified in the lower and lower-middle social classes in bivariate analysis (Table 1). It was also found in bivariate analysis that use of dental services for emergencies and/or dental pain was associated with low OHL, as well as tooth brushing less than twice daily, a greater prevalence of severe impact on OHRQoL, and presence of untreated caries (Table 1).

**Table 1:** Oral Health literacy according to sample characteristics, Piracicaba, Brazil, 2010

Variables		Low oral health literacy *n* (%)	High oral health literacy *n* (%)	Odds ratio (95% CI)	*p* value
Age (years)	45–64	83 (71.5)	27 (28.5)	1.84 (0.97–3.46)	0.061
	20–44	84 (62.7)	54 (37.3)	1	
Sex	Male	47 (68.3)	23 (31.7)	1.28 (0.64–2.50)	0.480
	Female	121 (73.2)	58 (26.8)	1	
Social class	Lower	30 (71.5)	8 (28.5)	4.71 (1.43–15.46)	**0.012**
	Lower middle	121 (74.8)	46 (25.2)	3.95 (1.84–8.49)	**0.001**
	Middle	16 (42.9)	27 (57.1)	1	
Service used	Public	39 (68.7)	16 (31.3)	0.71 (0.33–1.55)	0.378
	Insurance	41 (66.0)	17 (34.0)	0.63 (0.22–1.83)	0.383
	Private	85 (75.5)	46 (24.5)	1	
Service evaluation	Not Good	29 (85.9)	6 (14.1)	2.64 (0.97–7.16)	0.056
	Good	136 (69.8)	74 (30.2)	1	
Frequency of dental visits	Emergency	102 (81.8)	30 (18.2)	3.04 (1.60–5.80)	**0.001**
	Regularly	62 (59.7)	51 (40.3)		
Reason for the use of dental services	Pain	44 (85.4)	11 (14.6)	3.27 (1.56–6.82)	**0.003**
	Need	39 (75.1)	15 (24.9)	1.68 (0.66–4.27)	0.265
	Routine	78 (64.2)	53 (35.8)		
Tooth brushing	≤2 per day	68 (71.5)	21 (28.5)	2.12 (1.00–4.47)	**0.050**
	3+ per day	99 (66.4)	60 (33.6)	1	
Tooth flossing	irregularly	109 (71.5)	39 (28.5)	1.55 (0.84–2.89)	0.158
	regularly	58 (66.0)	42 (34.0)	1	
Impact on OHRQoL	Yes	89 (71.5)	26 (18.6)	2.65 (1.19–5.89)	**0.018**
	No	78 (62.3)	55 (37.7)	1	
Decayed teeth	Yes	69 (79.8)	22 (20.2)	1.95 (1.00–3.79)	**0.031**
	No	98 (67.0)	59 (33.0)	1	
Missing teeth	More than 13 teeth	45 (80.2)	13 (19.8)	0.55 (0.23–1.36)	0.188
	Up to 12, including anterior teeth	39 (56.7)	23 (43.3)	1.00 (0.41–2.47)	0.996
	Up to 12, including posterior teeth	42 (80.2)	15 (19.2)	0.32 (0.10–1.04)	0.058
	No	41 (69.0)	29 (31.0)		
Filled teeth	Yes	140 (70.0)	72 (30.0)	1.89 (0.74–4.81)	0.177
	No	27 (81.5)	9 (18.5)	1	
CAL > 4 mm	Yes	20 (25.6)	62 (74.4)	1.28 (0.66–2.52)	0.450
	No	105 (69.4)	61 (30.6)	1	
Dental plaque	Yes	77 (78.7)	24 (21.3)	1.90 (0.90–4.01)	0.090
	No	85 (66.1)	56 (33.9)	1	
Bleeding gum	Yes	86 (68.6)	45 (31.4)	1.36 (0.83–2.23)	0.214
	No	81 (74.8)	36 (25.2)	1	

*OHRQoL* Oral Health Related Quality of Life, *CAL* Clinical Attachment Loss, *CI* Confidence Interval

Bold numbers represent significant *p* values

Among the examined adults, 32.4% (*n* = 80) reported having no access to health information and 71.5% (*n* = 167) had low OHL. Question 1 “I can collect oral health-related information from various sources” and Question 4 “I can interpret and judge the credibility of the oral health information” were the least prevalent, thus indicating low OHL regarding communicative and critical health literacy (Table 2).

**Table 2:** Distribution of communicative and critical oral health literacy scale^a^ items, Piracicaba, Brazil, 2010 (*n* = 248)

Questions		n** (%)	95% CI
1	I can collect oral health-related information from various sources.	142 (53.7)	47.6–72.8
2	I can extract the information I want related to my oral health.	160 (64.5)	52.5–76.8
3	I can understand and communicate the oral health information obtained.	193 (77.8)	67.4–84.9
4	I can interpret and judge the credibility of the oral health information.	145 (58.5)	40.0–63.8
5	I can make decisions based on the information obtained and relate it to my situation and oral health issues.	172 (69.4)	55.6–72.4

CI = Confidence Interval

*Notes*: ^a^Oral Health Literacy questionnaire adapted from Ishikawa, 2008

**Number of participants who have agreed or strongly agreed that they have that ability

Low OHL was associated with only one oral health status measure after controlling for sex, age and SES in the logistic model 2 (Table 3). This measure was the presence of dental plaque (OR = 1.83, 95% CI = 1.08–3.33). In addition, having decayed teeth (OR = 1.92, 95% CI = 1.07–3.45) presented significant association with low OHL only when the odds ratio was adjusted for age and sex, in model 1 (Table 3).

**Table 3:** Oral health literacy as predictor of oral health outcomes among adults, Piracicaba, Brazil, 2011

Oral Health Outcomes	Oral health literacy	Model 1			Model 2		
Ref	Low	OR	95% CI	*p*-value	OR	95% CI	*p*-value
Decayed teeth^a^ No decayed teeth	Yes	1.92	1.07–3.45	**0.028**	1.6	0.92–2.79	0.096
Filled teeth No filled teeth	Yes	0.67	0.30–1.51	0.336	0.83	0.36–1.91	0.653
Tooth Loss^a^ No missing teeth because of oral disease	13 teeth or more	1.92	0.69–5.37	0.214	0.94	0.30–2.98	0.912
	Up to 12, including anterior teeth	1.01	0.45–2.25	0.976	0.55	0.22–1.37	0.201
	Up to 12, including posterior teeth	1.77	0.80–3.93	0.160	1.19	0.69–5.37	0.214
Periodontal Disease^a^ CAL <4 mm	CAL +4 mm	1.61	0.87–2.97	0.131	1.39	0.73–2.65	0.322
Dental plaque No biofilm	Yes	2.12	1.20–3.77	**0.010**	1.83	1.01–3.33	**0.047**
Bleeding gum No bleeding gum	Yes	0.93	0.53–1.55	0.713	0.87	0.49–1.53	0.622

Notes: ^a^OR significant for social class

Model 1 - adjusted by sex and age

Model 2 - adjusted by sex, age and social class

Regarding oral health practices, low OHL was associated with the use of dental services for emergency only (OR = 2.24, 1.24–4.04), with reason for use of dental services due to pain (OR = 2.21, 1.02–4.77) and with a dental service evaluation of ‘not good’ (OR = 2.61, 1.00–6.84), controlling for sex, age and social class/SES (Table 4). The study also found that a greater prevalence of oral health impacts on the quality of life (OR = 2.06, 1.15–3.69) presented a statistically significant association with low OHL even after adjustment for the effect of SES (Table 4). Tooth brushing <3 times/day (OR = 2.00, 1.11–3.62) and irregular tooth flossing (OR = 2.17, 1.24–3.80) were additional outcomes associated with low OHL, but these outcomes did not remain statistically significant once adjustment was made for SES (Table 4).

**Table 4:** Oral health literacy as predictor of personal health practices among adults, Piracicaba, Brazil, 2011

Health practices	Oral health literacy	Model 1			Model 2		
Ref	Low	OR	95% CI	*p*-value	OR	95% CI	*p*-value
Tooth brushing^a^ (3 times per day or more)	<2× per day	2.00	1.11–3.62	**0.022**	1.52	0.82–2.84	0.188
Tooth flossing^a^ (Regularly)	Irregularly	2.17	1.24–3.80	**0.007**	1.69	0.93–3.06	0.083
Frequency of use of dental care^a^ (Regularly)	Emergency	2.85	1.63–4.97	**<0.001**	2.24	1.24–4.04	**0.008**
Dental service evaluation (Good)	Not good	2.89	1.13–7.35	**0.026**	2.61	1.00–6.84	**0.050**
Reason for the use of dental services^a^ (Routine)	Pain	2.66	1.26–5.65	**0.011**	2.21	1.02–4.77	**0.045**
	Need	1.57	0.77–3.19	0.211	1.41	0.681–2.96	0.351
Impact on OHRQoL^a^ (OHIP prevalence^b^)	Yes	2.36	1.35–4.15	**0.003**	2.06	1.15–3.69	**0.015**

Note: ^a^OR significant for social class

Model 1 - adjusted by sex and age

Model 2- adjusted by sex, age and social class

^b^ OHIP prevalence means one or more impact often/very often

## Discussion

In Macek’s conceptual model of Health Literacy, we can note the importance of having this skill in making appropriate decisions regarding health, whether in the professionally recommended frequency of use of health services or in health-related behavior, which will impact health outcomes [21]. The low OHL in this study was associated with some oral health outcomes and oral health practices. There was a higher prevalence of adults with low OHL among those who used the emergency dental service, those who sought the service motivated by pain and those who evaluated the dental service used as not being good. Low OHL was also linked to the presence of dental plaque/biofilm and to greater impact of oral conditions on quality of life.

Despite all the communication resources existing today, especially with the advent of the Internet, many persons still do not use these resources and do not have access to health information. In Piracicaba, 32.4% of the adults in the research reported not having access to health information and the item of the questionnaire with the highest prevalence of low OHL was “I can collect oral health-related information from various sources.” This result brings with it the issue of where the gaps between the many pieces of health information in the media exist and how one accesses these pieces of information. Among the adults interviewed in this study, 71.5% presented low OHL; in the study of Apolinario et al. [22], conducted in Brazil, 66% of respondents presented inadequate health literacy.

Health literacy was highly associated with schooling in the study of Apolinario et al. [22]. The present study found the same association, but education level was measured and considered as part of the social class variable (SES), with few years of education grouped with lower SES, and lower SES being associated with low OHL. However, according to Apolinario et al. [22], the concept of health literacy (HL) does not end with the number of years studied. According to the authors, we cannot measure HL by the number of years studied, as they found 17% of individuals with appropriate HL among those who had low education. Kelly and Haidet [23] also discuss the importance of having appropriate tools to measure health literacy, because years of schooling alone as a measure, are not enough to identify patients who have greater difficulty understanding health information and who are able to make health decisions based on scientific evidence.

In our study, brushing and flossing regularly were not associated with OHL when adjusted for SES, but the presence of dental plaque was, showing the same anticipated relationship, that is, those who presented high OHL had lower prevalence of biofilm. Ueno et al. [24] have found an association between the level of oral health literacy and oral health behaviors and oral hygiene status, measured by the presence of dental plaque, as in our study. Ueno et al. [24] found that the higher the oral health literacy, the more frequently the patients brushed their teeth or dentures and the better their oral hygiene status. Lee et al. [25] also found that an increase in the oral health literacy was associated with better oral hygiene.

Adults who sought the dentist for routine visits also presented higher OHL according to Ueno et al. [24], thus corroborating the findings in our study, in which the prevalence of those who sought the dentist motivated by pain and used the service for emergency was greater among those with low OHL, as Parker and Jamieson [26] have also found among Aboriginal adults in Australia.

A systematic review of HL and health results found that individuals with low HL often have poor knowledge of health, poorer health and are less likely to use preventive services. In addition, they have higher rates of hospitalization and higher healthcare costs. Health literacy has been shown to be a mediator among health determinants, such as income, education and race, health behaviour and health outcomes [8].

Interestingly, Richman et al. [27] have reported that OHL was not associated with dental health status, but higher OHL scores were significantly associated with less perceived OHIP-14 impacts, which indicates better oral health-related quality of life (OHRQoL). According to Divaris et al. [28], HL presented inverted association with OHRQoL scores, which means that those with low health literacy had higher impacts on their OHRQoL, as in our study, in which adults who presented greater impact on their quality of life showed higher prevalence of low OHL. Even though caries and tooth loss were not associated with low OHL in our study once the analyses were adjusted for SES, these oral diseases can impact on the oral health-related quality of life of adults [12], thus demonstrating a relationship between OHL and the consequences of poor oral health using the subjective indicator of oral health impact on quality of life. Decayed teeth were associated with low OHL before adjustment was made for SES demonstrating the strength of the determinants of health for this outcome.

According to the conceptual model of Macek et al. [21], one’s health literacy and knowledge of health, modulated by socioeconomic and demographic variables, can generate appropriate oral health decisions that will impact on health outcomes. Health determinants such as income, education and personal characteristics influence health behaviours and oral health outcomes according to the conceptual model proposed. Moreover, HL is one of the strategies of health promotion according to Nutbeam [4]. The increase in health literacy has the potential to promote better decisions based on information, reduce health risks, increase prevention and well-being and increase transit through health systems, generating patient safety, patient care and quality of life [29].

Oral health literacy allows for the formulation of more appropriate health strategies, as the identification of individuals or communities with low OHL alerts us to the need for better communication to reach target audiences. According to Lee et al. [30], many patients and their families may have difficulties in reading printed educational materials. In addition, the authors emphasize that, in cases of very low OHL, communication problems can occur between professionals and target patients or communities and there should be a special focus on cultural adaptation of the scientific language so that we can generate information that reach individuals, thus generating increased health skills.

Our study is a novel one in the sense that it presents both OHL and oral health status data in Brazil where studies on OHL are just beginning. However, one of the limitations of our population-based study is that the OHL instrument used has not been validated in Brazilian Portuguese. At the time of our study, this instrument was the only one available for measuring communicative and critical health literacy and we have adapted it for oral health literacy. This study is a preliminary first step towards more rigorous validity and reliability testing that is needed to employ the new 5-item OHL instrument to measure critical and communicative oral health literacy in this population. It is possible that the OHL measure was not discriminative enough to show associations with oral health measures, such as dental caries, missing teeth or periodontal disease in this study. However, significant associations were found between OHL and oral biofilm, emergency dental visits and OHRQoL, adjusting for age, sex and social class. Oral plaque or biofilm is considered one of the main, direct causes or risk factors for oral disease, whereas emergency dental visits are a consequence of poor oral health. Presence of plaque is also an oral health outcome as it is an indicator of poor oral hygiene practices. Furthermore, emergency dental visits and OHRQoL are considered ‘true’ endpoints for oral health, while the DMFT index is a ‘surrogate’ endpoint. Further studies should consider different ways to analyse OHL and improve OHL indicators.

Another potential study limitation was the higher percentage of respondents who were women. This higher representation of women can be explained by the fact that this was a household survey. In the National Oral Health Survey in Brazil (SB Brasil, 2010) [19], that has used the same study design, there was also a greater percentage of woman in the survey. This fact reflects the Brazilian cultural context where women stay at home more than men and are therefore are more likely to participate in health services research.

The results of our study allow us to know that, in addition to health determinants, OHL can be an intermediate factor that impacts on oral health outcomes, health behaviours and use of dental services. The relevance of this fact is that OHL can be changed with health promotion strategies, in this way positively influencing oral health outcomes and health practices, while macro-determinants are structurally more difficult to change.

## Conclusion

Oral health literacy is related to oral health status (biofilm) and practices, such as the use of emergency dental services, and could interfere with perceived impacts on quality of life. As low oral health literacy can be overcome, the results support oral health promotion strategies directed to improve oral health literacy in adult populations. Further studies should be conducted on OHL in order to understand the pathways by which OHL affects oral health.
