# Correction to: Oral health literacy and oral health outcomes in an adult population in Brazil

**DOI:** 10.1186/s12889-017-4827-1

**Published:** 2017-10-18

**Authors:** Marília Jesus Batista, Herenia Procopio Lawrence, Maria da Luz Rosário de Sousa

**Affiliations:** 1 Community Health, Jundiai Medical School, Francisco Telles, 250, P.O. Box1295, Jundiaí, SP 13202-550 Brazil; 2 Community Health Graduate Program, Piracicaba Dental School, University of Campinas, Avenida Limeira 901, P.O. Box 52, Piracicaba, SP 13414-018 Brazil; 3 Dental Public Health Discipline, Faculty of Dentistry, University of Toronto, Canada, 124 Edward Street, Toronto, ON M5G 1G6 Canada; 4 Department of Community Dentistry, Piracicaba Dental School, University of Campinas, Brazil, Avenida Limeira 901, P.O. Box 52, Piracicaba, SP 13414-018 Brazil

## Correction

After publication of the article [1], it has been brought to our attention that the publication  funding acknowledgement is missing from the original article. It should also include reference to grant number 2017/13264–7 from the Sao-Paulo Research Foundation.
